# Supplementary figures and images for: AlphaFold2-multimer guided high-accuracy prediction of typical and atypical ATG8-binding motifs
Source: PLoS Biol. 2023 Feb 8;21(2):e3001962. doi: 10.1371/journal.pbio.3001962 (PMC9907853; doi:10.1371/journal.pbio.3001962)

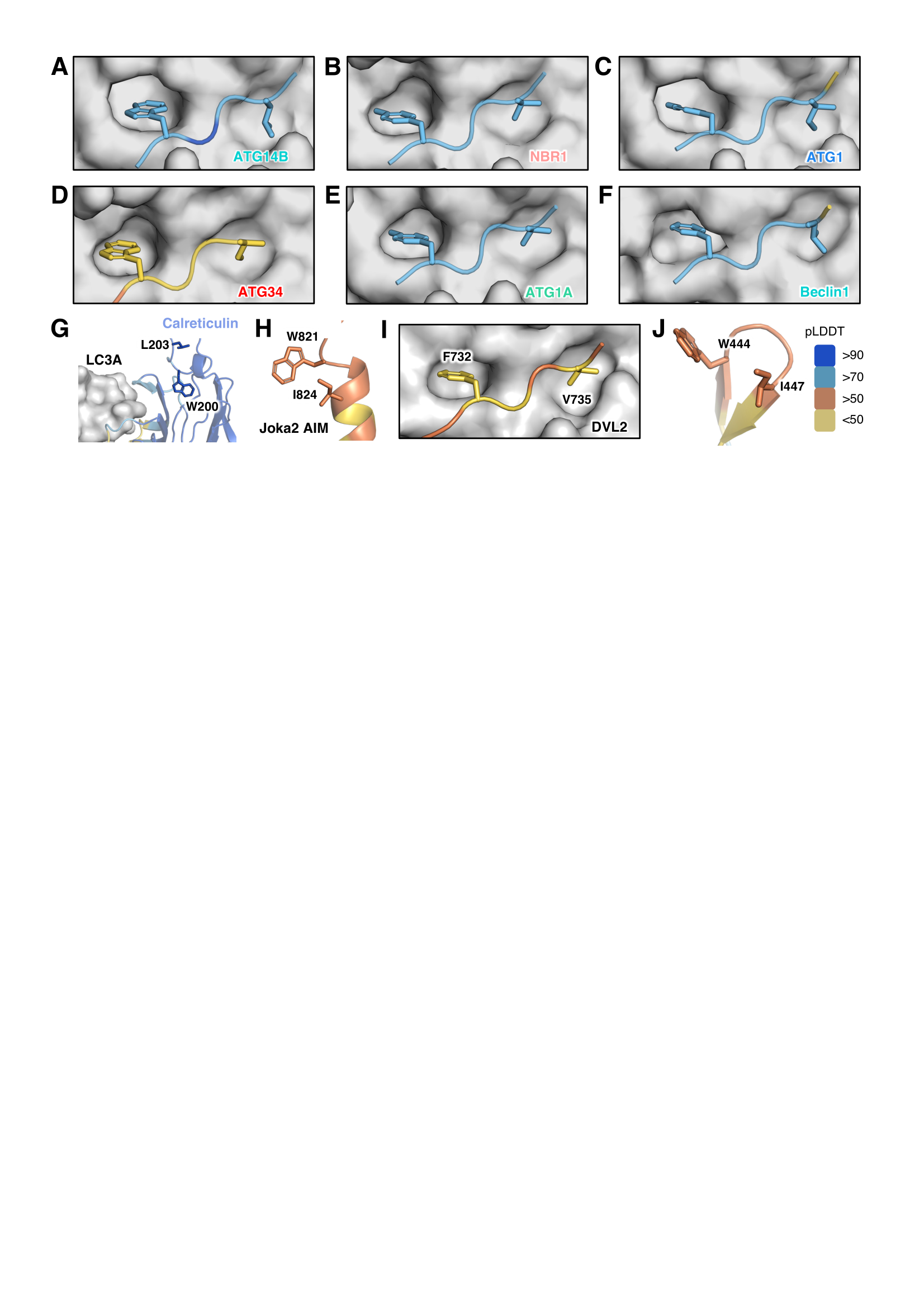

Supplement: S1 Fig — (A–F) AF2 and experimentally verified canonical AIM/LIR ATG8-family interactions, shown in Fig 1C–1H, coloured based on AF2-calculated prediction score; pLDDT. Blue indicates regions of a protein with a score of over 90, meaning a very high confidence prediction. Scores between 70 and 90 are represented with light blue, which accounts for a high confidence score. Scores between 50 and 70 are considered low (orange) and anything below 50 is a very low confidence prediction (yellow). (G) Calreticulin does not interact with LC3A via its canonical LIR, which seems to be positioned away from LC3A (surface representation–grey). However, Calreticulin may interact with the C-terminal region of LC3A. (H) Joka2 AIM residue ILE824 is localised in the alpha-helix and no ATG8 interaction is observed. (I) DVL2 may interact with LC3A LIR pockets via a different canonical AIM, (J) as the experimentally studied LIR was observed away from LC3A and structured into a beta-sheet. Calreticulin, Joka2, and DVL2 are coloured based on the AF2-calculated prediction confidence score, pLDDT. (TIFF) [file pbio.3001962.s001.tiff]

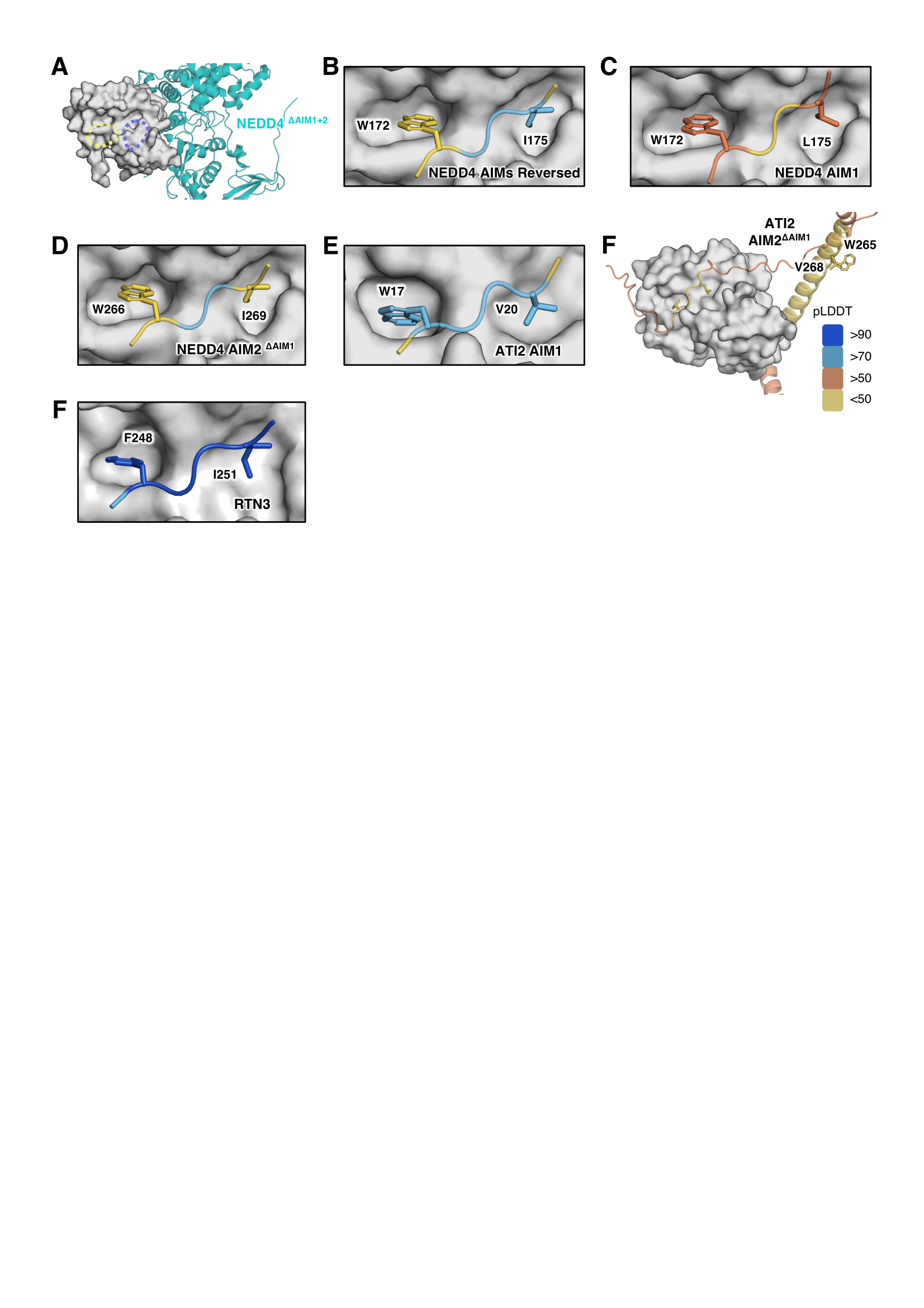

Supplement: S2 Fig — (A) The pockets of LC3B remain empty when both LIRs of NEDD4 are truncated. (B) Upon the reversal of LIR -1 and -2 positions, AF2-multimer favours LIR1 (previously LIR2). (C, D) AF2-multimer prediction confidence of the NEDD4 AIM1 and AIM2 in complex with LC3B. (E) AF2-multimer prediction confidence for ATI2 AIM1 in complex with ATG8CL. (F) ATI2 AIM2 cannot occupy ATG8CL pockets when AIM1 is truncated. (G) Predicted GABARAP and RTN3 interaction by LIR3; FEVI. NEDD4 and ATI2 in panels (B–F) are coloured based on the AF2-calculated prediction confidence score; pLDDT. Blue indicates regions of a protein with a score of over 90, meaning a very high confidence prediction. Scores between 70 and 90 are represented with light blue, which accounts for a high confidence score. Scores between 50 and 70 are considered low (orange) and anything below 50 is a very low confidence prediction (yellow). (TIFF) [file pbio.3001962.s002.tiff]

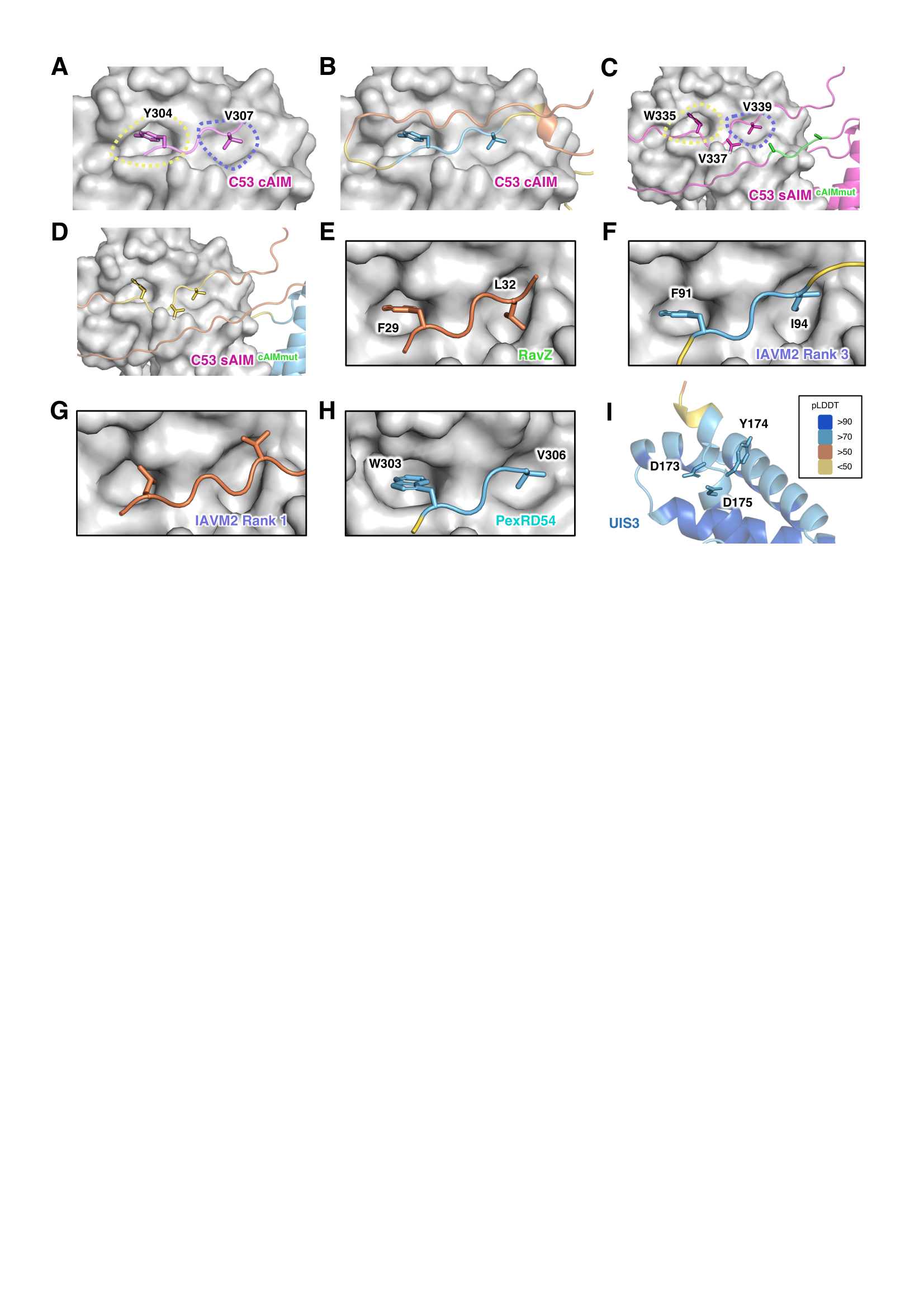

Supplement: S3 Fig — (A, B) Wild-type C53 preferentially interacts with ATG8A pockets via the canonical AIM (cAIM) formed by (304)YxxV(307) and this region of the protein within the complex is predicted at a high confidence. (C) Once the canonical AIM (cAIM—green) of C53 (pink) is mutated, a complex with ATG8A is formed by a shuffled AIM (sAIM); (F) however, this is not at high confidence, although it matches experimental observations. (E) RavZ, (F) IAVM2 model ranked third, with highest confidence at LIR region, (G) rank one model for IAVM2 and (H) PexRD54 complexes with respective ATG8-family members. (I) The suggested LIR of UIS3 (DYD) resides in an alpha-helix. All panels are coloured based on the AF2-calculated prediction confidence score; pLDDT. Blue indicates regions of a protein with a score of over 90, meaning a very high confidence prediction. Scores between 70 and 90 are represented with light blue, which accounts for a high confidence score. Scores between 50 and 70 are considered low (orange) and anything below 50 is a very low confidence prediction (yellow) (see Methods for further details). (TIFF) [file pbio.3001962.s003.tiff]

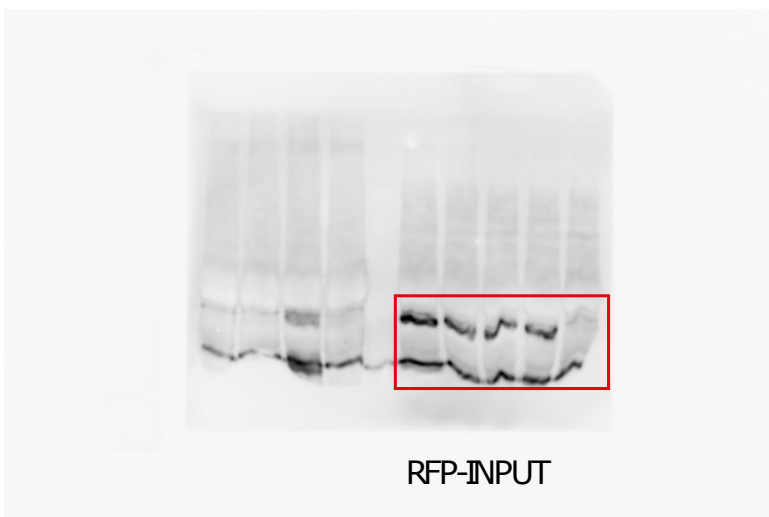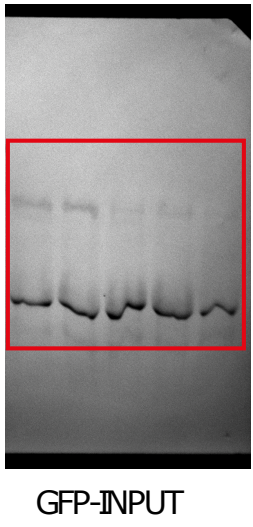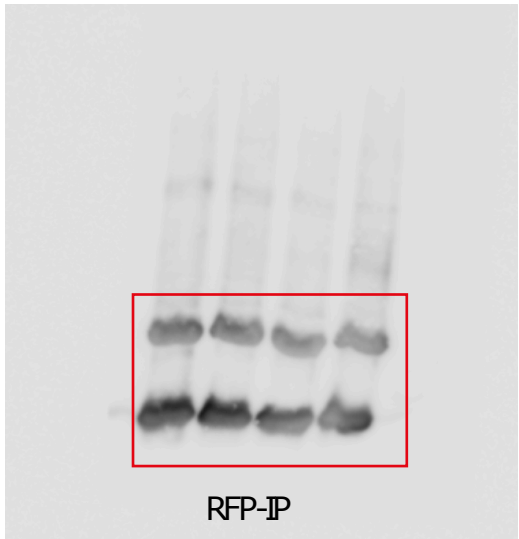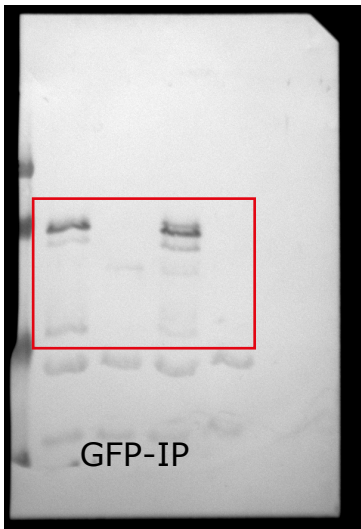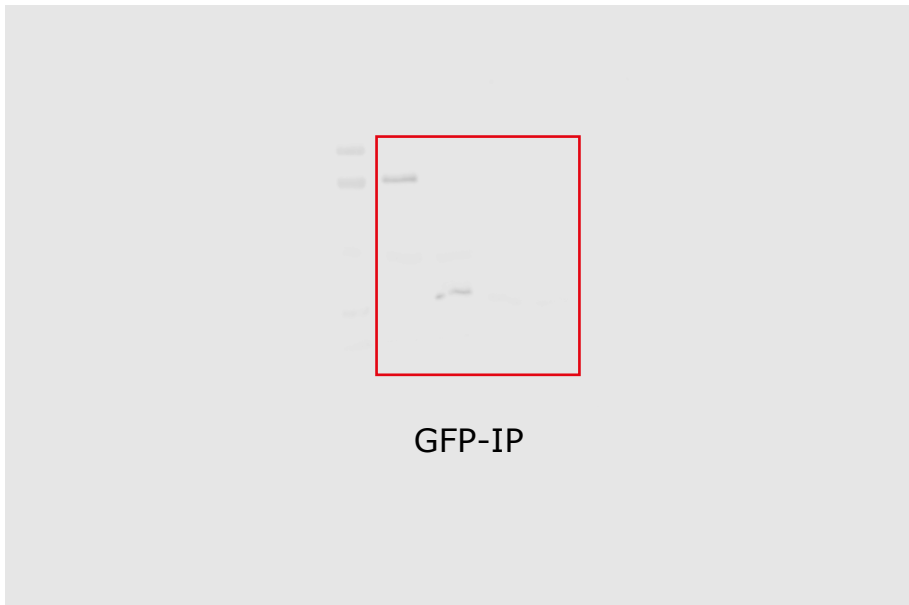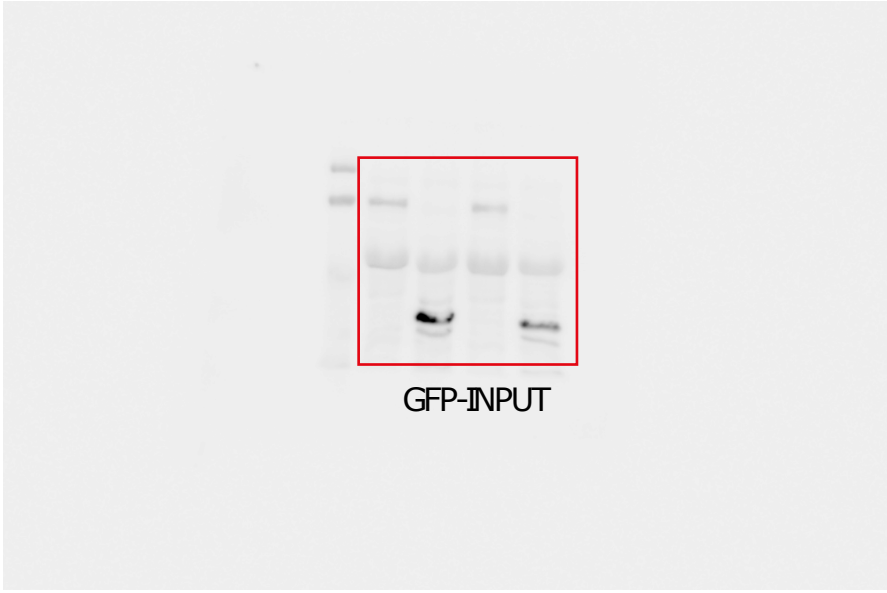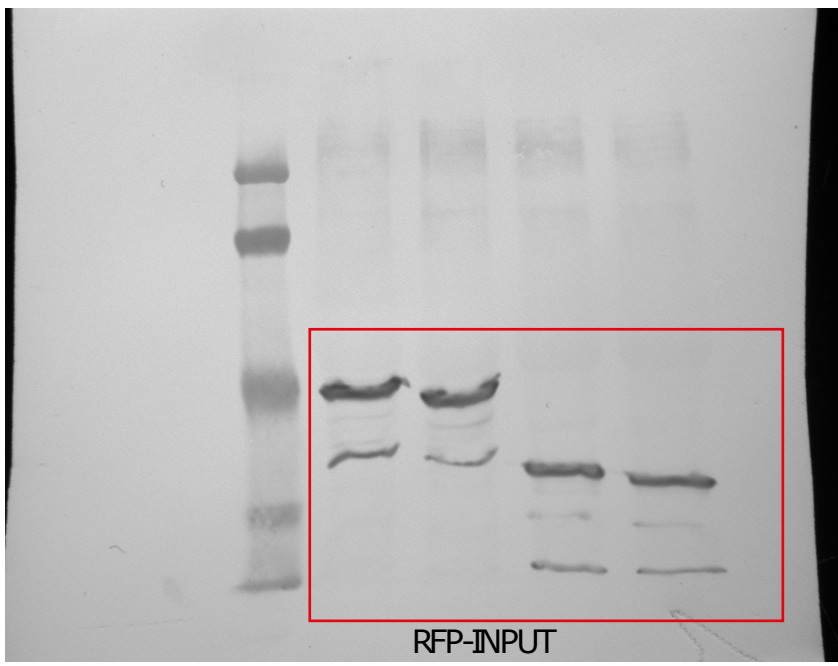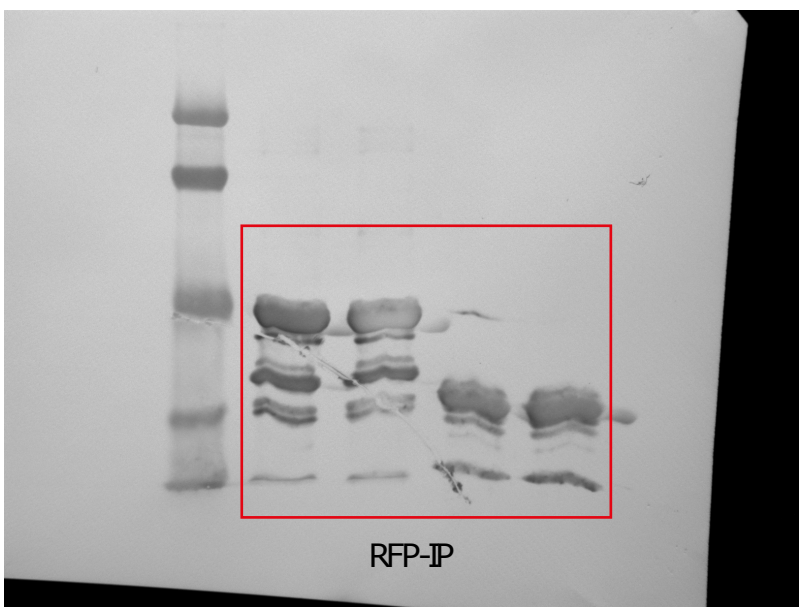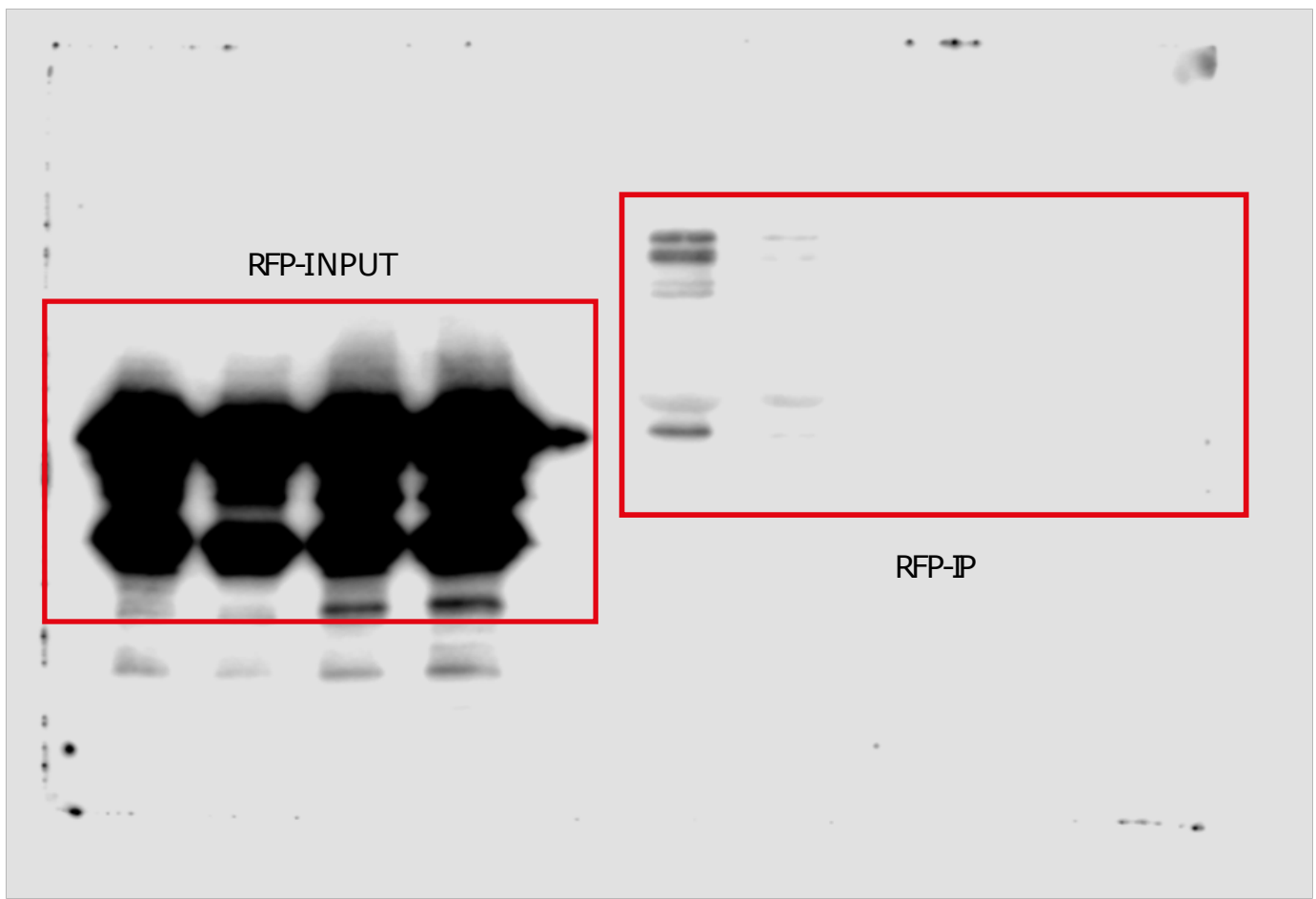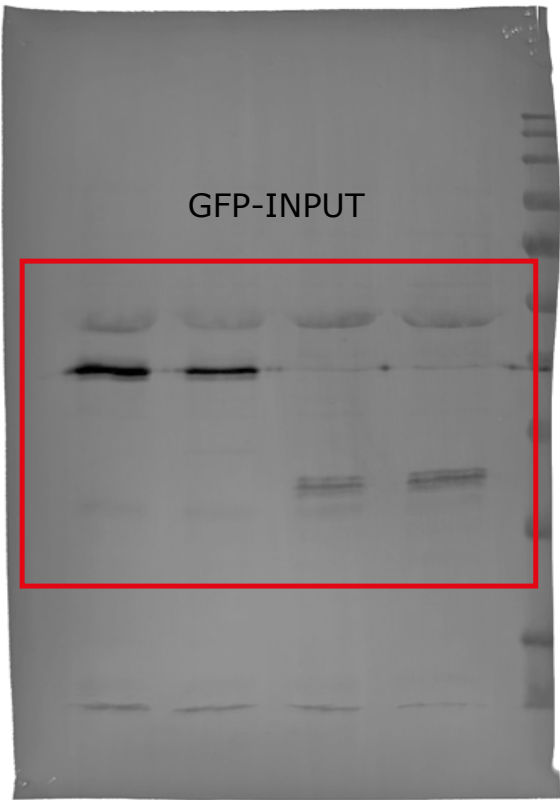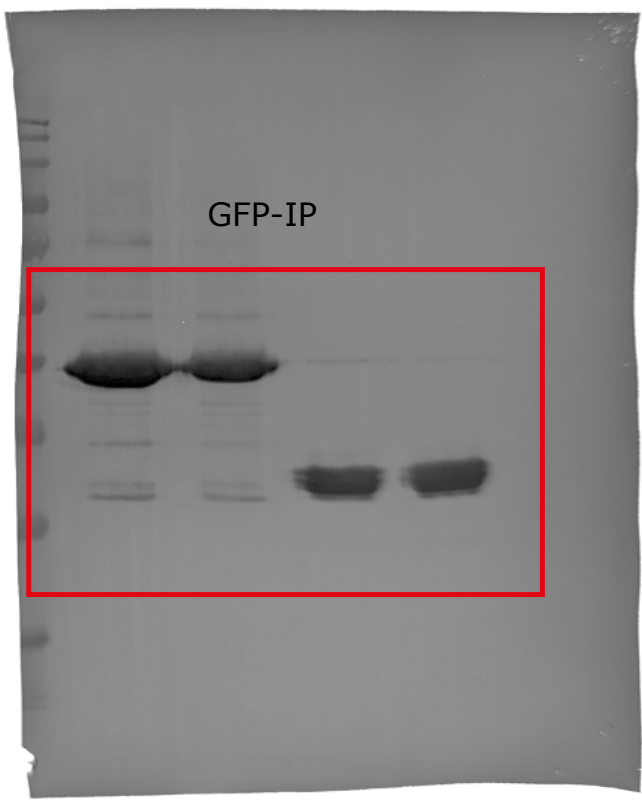

Supplement: S1 Raw images — (PDF) [file pbio.3001962.s007.pdf]
