# Supplementary material for: AlphaFold2-multimer guided high-accuracy prediction of typical and atypical ATG8-binding motifs
Source: PLoS Biol. 2023 Feb 8;21(2):e3001962. doi: 10.1371/journal.pbio.3001962 (PMC9907853; doi:10.1371/journal.pbio.3001962)
Supplement: S3 Data — (RTF) [file pbio.3001962.s006.rtf]

  .y.   group1 group2        p   p.adj p.format p.signif method  
  <chr> <chr>  <chr>     <dbl>   <dbl> <chr>    <chr>    <chr>   
1 puncta WTAIMp Maimp  0.000241 0.00024 0.00024  ***      Wilcoxon
