# Supplementary material for: AlphaFold2-multimer guided high-accuracy prediction of typical and atypical ATG8-binding motifs
Source: PLoS Biol. 2023 Feb 8;21(2):e3001962. doi: 10.1371/journal.pbio.3001962 (PMC9907853; doi:10.1371/journal.pbio.3001962)
Supplement: Attachment — Submitted filename: ReviewerReplies_FINAL.docx [file pbio.3001962.s008.docx]

**AlphaFold2-multimer guided high accuracy prediction of typical and atypical ATG8 binding motifs**

**Ibrahim et al.**

**Response to reviewers**

We are writing in response to your e-mail message with peer-review comments on our manuscript “AlphaFold2-multimer guided high accuracy prediction of typical and atypical ATG8 binding motifs” (PBIOLOGY-D-22-02166R1). We were pleased and encouraged by the reviewers’ recognition of the interest and significance of our results. We thank the reviewers for their suggestions on how we could strengthen and improve the manuscript. We have now revised our manuscript to address the reviewers’ concerns, as detailed below.

***REVIEWER COMMENTS***

*Editorial and data-related requests:*

*(A) You may be aware of the PLOS Data Policy, which requires that all data be made available without restriction: http://journals.plos.org/plosbiology/s/data-availability. For more information, please also see this editorial: http://dx.doi.org/10.1371/journal.pbio.1001797*

*Note that we do not require all raw data. Rather, we ask that all individual quantitative observations that underlie the data summarized in the figures and results of your paper be made available in one of the following forms:*

*- Supplementary files (e.g., excel). Please ensure that all data files are uploaded as 'Supporting Information' and are invariably referred to (in the manuscript, figure legends, and the Description field when uploading your files) using the following format verbatim: S1 Data, S2 Data, etc. Multiple panels of a single or even several figures can be included as multiple sheets in one excel file that is saved using exactly the following convention: S1_Data.xlsx (using an underscore).*

*- Deposition in a publicly available repository. Please also provide the accession code or a reviewer link so that we may view your data before publication.*

All AF2-multimer prediction folders involving proteins mentioned in Supplemental Table 1 have been uploaded to the public repository **figshare** and is available at <https://doi.org/10.6084/m9.figshare.21533769.v1>. This link has been added to relevant figure legends.

*Regardless of the method selected, please ensure that you provide the individual numerical values that underlie the summary data displayed in the following figure panels as they are essential for readers to assess your analysis and to reproduce it:*

*Figure 3G*

*NOTE: the numerical data provided should include all replicates AND the way in which the plotted mean and errors were derived (it should not present only the mean/average values).*

Individual numerical values summarised in Figure 3G are available at S2_Data.csv and the R-studio code used for the statistical analysis can be found in S3_Data.rtf.

*(B) Please also ensure that each of the relevant figure legends in your manuscript include information on *WHERE THE UNDERLYING DATA CAN BE FOUND*, and ensure your supplemental data file/s has a legend.*

Main text figure legends have been updated to refer to the supplementary information provided. The accession link for AF2-multimer data has also been added.

*(C) We require the original, uncropped and minimally adjusted images supporting all blot and gel results reported in the following figures:*

*Figure 2G-H, 3F*

Uncropped blot images have been made available under S1_Raw_Images.pdf. We noticed that we miss labelled the wrong sized band in Figure 2G panel 3, and corrected this in the figure.

*We will require these files before a manuscript can be accepted so please prepare and upload them now. Please carefully read our guidelines for how to prepare an and upload this data: https://journals.plos.org/plosbiology/s/figures#loc-blot-and-gel-reporting-requirements.*

*Reviewer #1:*

*In this manuscript, Ibrahim et al. examined the utility of the protein structure prediction tool AlphaFold-Multimer (AF2-multimer) to identify ATG8-interacting motifs (AIMs) respective LC3-interacting regions (LIRs). Using a set of nearly three dozen well-characterized ATG8/LC3-interacting proteins the authors showed that AF2-multimer is able to detect the vast majority of established canonical and non-canonical AIM/LIRs. By combining in silico motif-swapping and -deletion approaches with biochemical experiments on the plant protein ATI2 the authors confirmed that AF2-multimer correctly maps canonical AIMs/LIRs among several potential candidates. The authors went on to showed that this approach also allows the identification of a previously uncharacterized, non-canonical-type AIM in plant ATG3. Lastly, the authors expanded their analysis to ATG8/LC3-binding virulence factors from different human and plant pathogens and found that AF2-multimer correctly predicted their experimentally validated AIMs/LIRs. Overall, the work of Ibrahim and colleagues elegantly shows that AF2-multimer cannot only be employed to identify validated AIMs/LIRs but might also be a useful tool to map new ATG8/LC3 binding sites. However, a few critical issues remain.*

*1) While the authors show AF2-multimer can identify functional AIM/LIR in proteins with up to two AIMs/LIRs candidates (e.g., NEDD4 and ATI2), a number of proteins have far more LIRs than that. The authors should examine AF2-multimer's performance on proteins with truly multiple AIMs/LIRs such as RTN4 (Grumati et al. Elife 2017 (PMID: 28617241)).*

To test the ability of AF2-multimer in the identification of AIM/LIR proteins with more than two candidates, we investigated human RTN3 (hRTN3) which reports six LIR motifs (PMID: 28617241). Running AF2-multimer with hRTN3 showed a canonical LIR at the third position (FEVI), in agreement with structural data (Supplemental Figure 2G). *In silico* truncation of this LIR, did not show any additional LIR interactions with its partner GABARAP-L1. Interestingly, only this LIR is conserved, which may indicate a limitation of our phylogenetic-based AI approach.

*2) To test the limits/capabilities of AF2-multimer, the authors should examine the non-canonical LIR in ATG12 (Kaufmann et al. Cell 2013 PMID: 24485455).*

To test the limitations and capabilities of AF2-multimer, we next explored whether the noncanonical LIR in ATG12 could plausibly bind ATG8 (PMID:24485455). Although AF2-multimer did not predict any AIM-dependent interaction between these two proteins, a possible additional interaction interface was highlighted, which requires further investigation.

*3) All immunoblots are missing molecular weight markers. Please add them.*

All blot figure panels have been updated with appropriate molecular weight markers.

*Reviewer #2:*

*Ibrahim et al have used the AlphaFold2-multimer software tool to perform protein modelling to predict LIR/AIM motifs in proteins bidnign to ATG8 family proteins.*

*I was amazed how succesful the authors have been using this tool to predict LIR motifs of both known and unknown LIR-containing proteins and also of non-canonical LIR motifs.*

*This is a really large step forward in predicting ATG8-interacting motifs that the autophagy research community will benefit from!*

*It is also of general interest as a strategy and tool to find motifs binding to specific proteins.*

*I am very positive to this nice work and do not have major criticisms.*

*The Introduction does not mention ATG8ylation and could perhaps refer to a review on this (PMID: 34671813, PMID: 34527862).*

This reference is now added (20, 21)

“Therefore, identifying and characterizing the AIM/LIR residues has been a crucial step in dissecting the molecular basis of autophagy regulation from initiation to selective cargo sorting and autophagosome transport, which may also be important for understanding newly discovered ATG8ylation process (PMID: 34671813, PMID: 34527862).”

*It would perhaps also be pertinent to refer to an extensive review on ATG8s and LIR motifs (PMID: 31310766) in the second paragraph of the INTRODUCTION.*

This reference is now included (19).

*It was not clear to me the exact sequences used to do the modeling and prediction in each case. This needs to be shown in supplemental material.*

Data including exact sequences used to do the AF2-multimer modelling and prediction for each case is available in S1_Data.xlsx.

*I did not see a supplemental Table 1 referred to in the paper, only an excel file with core LIR sequences.*

Data supporting Fig 1-4 available in S1_Data.xlsx and called out in each legend. Any reference to Supplemental Table 1 has been removed.

*It is important to know if a 300 amino acid sequence can be placed in and give an accurate prediction of one LIR sequence for example or if shorter sequences must be probed sequentially to get the best results.*

In our test sets, we investigated the ability of AF2-multimer to predict structural interactions of proteins comprised of up to 1478 residues.
